# The Application of Integrated Force and Temperature Sensors to Enhance Orthotic Treatment Monitoring in Adolescent Idiopathic Scoliosis: A Pilot Study

**DOI:** 10.3390/s25030686

**Published:** 2025-01-23

**Authors:** Yiying Zou, Lejun Zhou, Jinhao Wang, Edmond Lou, Man-Sang Wong

**Affiliations:** 1 Department of Biomedical Engineering, The Hong Kong Polytechnic University, Hong Kong, China; yiying-jojo.zou@connect.polyu.hk (Y.Z.); lejun-casper.zhou@polyu.edu.hk (L.Z.); 2 Department of Health Sciences and Technology, ETH Zurich, 8092 Zurich, Switzerland; jinhao.wang@hest.ethz.ch; 3 Department of Electrical and Computer Engineering, University of Alberta, Edmonton, AB T6G 1H9, Canada; elou@ualberta.ca

**Keywords:** adolescent idiopathic scoliosis (AIS), orthotic treatment, compliance, wearing pattern, temperature sensor, force sensor

## Abstract

Orthosis-wearing compliance is crucial for achieving positive treatment outcomes in patients with adolescent idiopathic scoliosis (AIS), for whom 23 h of daily wear is typically prescribed. However, self-reported compliance is subjective and often based on patients’ memory, leading to inaccuracies. While portable electronic devices have been developed to objectively monitor compliance, relying solely on temperature or force data can be insufficient. This study introduced a novel method that integrated both force and temperature data to estimate orthosis-wearing compliance. Twelve patients (eight females and four males) diagnosed with moderate AIS were included. Each patient was prescribed a thoracic-lumbar-sacral orthosis equipped with an integrated force and temperature sensor system. After one month of orthotic treatment, self-reported wear time averaged 17.8 ± 6.2 h/day, while the sensor indicated an average wear time of 13.3 ± 5.0 h/day. Most patients overestimated their compliance. Nighttime was the most common period for orthosis wear (6.1 h/day), whereas compliance during school hours (2.8 h/day) and after-school hours (3.7 h/day) was lower. The integration of force and temperature sensors provides a more comprehensive understanding of orthosis compliance. Future studies with larger samples and longer monitoring periods are needed to investigate the correlation between compliance and treatment outcomes.

## 1. Introduction

Adolescent idiopathic scoliosis (AIS) is the most common spinal deformity in adolescents, affecting approximately 1–4% of the global adolescent population [1]. Diagnosis is typically made using the Cobb angle, which is measured from the standing radiographs [2]. Left untreated, AIS may lead to severe physical deformity, affected aesthetics, back pain, compromised cardiopulmonary function, and reduced quality of life [3,4,5]. Orthotic treatment is a recommended conservative method when the Cobb angle is between 20 and 45 degrees, particularly during a patient’s growth spurt [6]. Full-time orthotic treatment requires wearing the orthosis for 23 h daily until skeletal maturity is reached. However, the effectiveness of bracing is highly dependent on patient compliance. Studies have shown that higher and more consistent compliance with orthotic treatment is associated with reduced curve progression and a decreased likelihood of spinal surgery [7,8]. Traditionally, compliance has been determined through patient and family reporting, along with visual inspection of the orthosis for wear and tear [9,10]. Self-reported compliance is subjective, prone to recall bias, and unable to precisely capture wearing habits. To overcome these limitations, objective monitoring tools such as temperature and force sensors have been developed.

Temperature sensors, which detect heat at the skin–orthosis interface, assume wear based on thresholds typically ranging from 28.0 °C to 36.7 °C [11,12,13,14,15]. Different studies may choose slightly different thresholds within this range (e.g., 28 °C, 29 °C, 30 °C). However, in regions with prolonged hot seasons and high ambient temperatures, environmental temperatures can often exceed these thresholds, leading to false detections [16,17]. As a result, relying solely on temperature sensors risks inaccurate measurements, as high ambient temperatures may be misinterpreted as orthosis wear. On the other hand, force sensors have been employed to monitor compliance by measuring the pressure exerted by the orthosis on the trunk, including wearing tightness. Previous studies typically used fixed thresholds, ranging from 0 N to 7.9 N, to estimate wearing compliance [16,18,19,20,21]. While one study used a threshold of 0 N to identify any pressure as a wear event [22], another study found that force readings could drop to zero during self-correction exercises or due to a loose orthosis fit, even when the orthosis was still being worn [18]. Compared to estimating wear time, force-based monitoring may perform better in assessing how tightly the orthosis is worn, but it still has limitations that can lead to an underestimation of wear time [16]. These limitations highlight the need for more comprehensive monitoring methods of compliance.

To address these limitations, there is a growing interest in multi-parameter monitoring systems that integrate temperature and force sensors. These systems utilize the complementary strengths of both modalities: temperature data provide insights into wear status, while force data capture variations in fit and pressure dynamics. By combining these parameters, it can better distinguish true orthosis wear from external factors, such as ambient heat or loose fitting. This study aims to evaluate the feasibility and preliminary accuracy of an integrated force and temperature sensor system for monitoring orthotic compliance in the patients with AIS.

## 2. Materials and Methods

This prospective observational pilot study was conducted on patients recruited from a large community scoliosis screening program. The study was approved by the local ethics committee (HSEARS20221012006). Written informed consent was obtained from all participants and their parents or guardians.

### 2.1. Compliance Monitoring System

**Figure 1:**
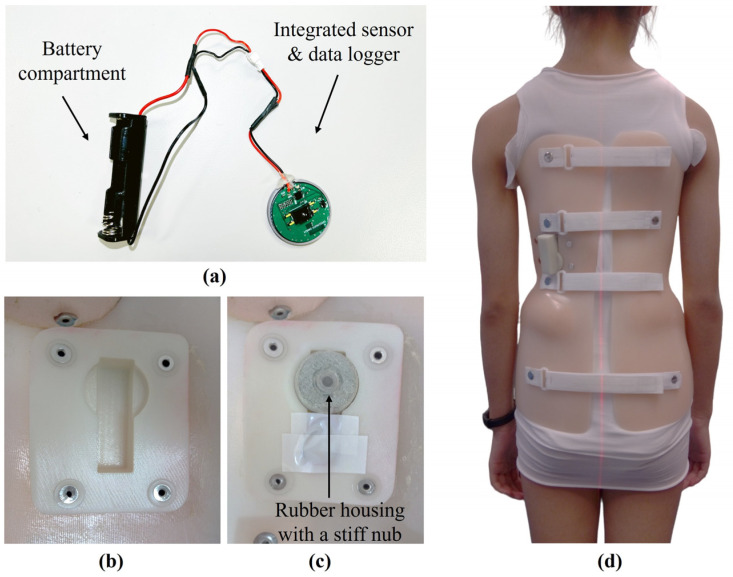
Components and application of the compliance monitoring system in an orthosis. (**a**) Battery compartment and the integrated sensor and data logger; (**b**) internal housing for securing the monitoring system within the orthosis; (**c**) housing with the monitoring system; (**d**) patient wearing the orthosis fitted with the compliance monitoring system, which was installed in the major correction area.

The compliance monitoring system consists of a battery-powered data logger (3 cm in diameter and 0.8 cm in thick) with integrated force and temperature sensors (Figure 1a). The system is housed in a rubber casing that includes a stiff nub over the force-sensing area to direct force onto the sensor, ensuring accurate measurements, as shown in Figure 1c. The force sensing module employs a FS1500 force sensor (Honeywell International Inc., Charlotte, NC, USA), which has a sensing range of 14.7 N and a sensitivity of 315 mV/N. The data logger is built on the CC2530F256 system-on-chip (Texas Instruments Inc., Dallas, TX, USA), which integrates an 8051 microcontroller, a radio transceiver with the wireless frequency of 2.4 GHz under the Institute of Electrical and Electronics Engineers (IEEE) 802.15.4 standard, and an onboard temperature sensor with an accuracy of ±1.5 °C, as specified by the manufacturer.

The integrated force and temperature sensor was manufactured in-house. The performance and reliability of the force sensor module were tested using a class-one level system in previous studies [16,23], where the force sensing module was evaluated under controlled laboratory conditions. The sensor was tested at three different temperatures (25 °C, 30 °C, and 35 °C), and the results showed no difference in the sensor response at different temperatures. The sensor demonstrated excellent performance metrics, including accuracy (1.9%), repeatability (0.6%), linearity (0.7%), hysteresis (0.8%), and reproducibility (1.3%). A calibrated load cell was used as the reference standard during testing. For this study, the force readings were digitized with a range of 10 N and a resolution of 0.05 N, as 10 N was determined to be the maximum expected force within the orthosis based on prior experience.

The device was positioned in the major correction area of the orthosis, covered with a pad, where the brace applied controlling forces to control spinal deformity, enabling accurate force monitoring (Figure 1b–d). It recorded force and temperature simultaneously at the skin–orthosis interface every minute for one month. The battery and memory capacities could last for more than 6 months when the 1 min per sample acquisition rate was used. Data were wirelessly downloaded using custom mobile software for analysis.

### 2.2. Participants

The inclusion criteria were as follows: (1) a diagnosis of AIS; (2) a primary Cobb angle between 20 and 40 degrees [6]; (3) age between 10 and 15 years; (4) for females, within one year of menarche; (5) no prior treatments for AIS. Exclusion criteria included secondary scoliosis, neuromotor disorders, psychological issues affecting compliance, or reluctance to undergo orthotic treatment.

Each patient was prescribed a Hong Kong-style orthosis, a thoracic-lumbar-sacral orthosis (TLSO) equipped with the monitor system. Patients were instructed to wear the orthosis for 23 h daily, but it was recommended to gradually increase the wearing time over the first month. Specifically, patients were advised to begin with 8 h per day, increasing the wear time by around 4 h each week, to reach around 23 h per day by the end of the first month. The first month served as an adaptation period, allowing patients to get used to the feeling of wearing the orthosis. Tailored undergarments were provided to each patient to protect the skin from direct friction and to ensure that the sensor operated in a consistent environment (same material and same thickness) across all patients. After the one-month adaptation period, patients returned to the clinic for a follow-up visit. The sensor data were retrieved at this visit, and the wearing compliance was evaluated.

### 2.3. Compliance Measurement

The force and temperature thresholds used to determine compliance were established through a preliminary test. Four patients participated in a laboratory-based preliminary test to establish thresholds for detecting orthosis compliance. The orthotist adjusted each patient’s orthosis to the prescribed level while the patients were in a standing position [24]. Afterwards, they remained in a controlled environment (ambient temperature: 24 ± 1 °C). Sensor data collected during this period were analyzed to establish baseline temperature and force fluctuation values. Given that a thick pressure pad covered the sensor, the recorded temperatures were expected to be lower than the actual skin surface temperature during wear. Additionally, the baseline force reading was anticipated to be above zero due to the pad’s pressure.

Data from the four patients showed that when the orthosis was worn, the sensor detected temperatures ranging from 28.4 °C to 31.1 °C. A consistent increase above 28 °C was observed during the “worn” status. To validate this threshold, the stabilization time of the temperature sensor was evaluated. Patients were instructed to wear or remove the orthosis in the laboratory, and continuous temperature readings showed that the sensor required approximately 30 min to stabilize and accurately reflect transitions between “worn” and “not worn” states.

Additionally, the force threshold was determined by the fluctuation frequency, calculated by analyzing consecutive minute-by-minute force values. A fluctuation was defined as a change in force exceeding the target force individually set for each patient. The fluctuation frequency during continuous orthosis wear ranged from 0.8 to 0.85 times per minute. Based on this analysis, 0.8 fluctuations per minute were selected as the threshold for the “worn” state, reflecting the natural force variation caused by body movements (breathing) while wearing the orthosis.

Building on the findings from the preliminary test, an algorithm was developed to calculate orthosis-wearing compliance based on the sensor data. The algorithm identified orthosis-wearing episodes when the sensor recorded temperatures above 28 °C, along with a force fluctuation frequency greater than 0.8 times per minute.

**Figure 2:**
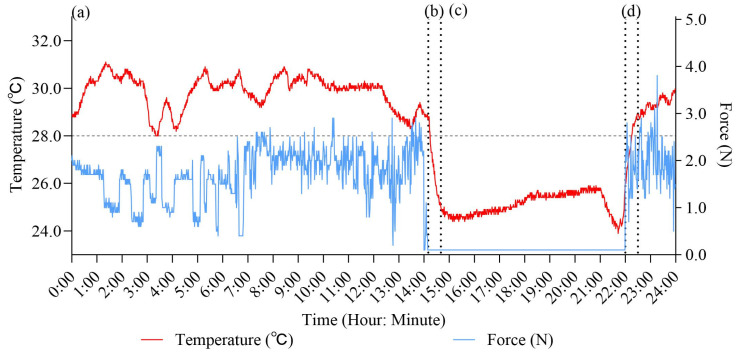
Typical changes in temperature (red) and force (blue) detected by sensors during different orthosis-wearing states. (**a**) Orthosis worn: both temperature and force increase significantly and exhibit fluctuations within a narrow range. (**b**) Orthosis taken off: temperature rapidly drops by several degrees, while force decreases sharply, almost 0 N. (**c**) Orthosis not worn: Temperature stabilizes at lower levels, close to room temperature. Any increases in room temperature or external heating would be reflected in changes to the temperature curve. Force remains stable at around 0 N. (**d**) Orthosis being put on again: temperature rises rapidly, and force begins to fluctuate.

To validate the threshold and capture sensor parameters during actual use, the patient and their parents or guardians assisted in recording the exact times of orthosis wear and removal one day before the follow-up visit. Limiting the recording to one day helped minimize the recording burden. These manual records were used as the ground truth for comparison with the sensor algorithm. During this one-day pilot study, the algorithm achieved an average accuracy of 92.3% in distinguishing between “worn” and “not worn” states across all participants. Figure 2 presents the typical temperature and force change patterns observed when the orthosis was worn (Figure 2a), taken off (Figure 2b), not worn (Figure 2c), or put on again (Figure 2d). Furthermore, during orthosis wear, the force readings exhibited a fluctuating pattern, whereas the force remained stable in non-wearing conditions.

### 2.4. Statistical Analysis

Compliance was measured both subjectively through patient self-reports and objectively using sensor data. To compare orthosis-wearing time across different periods, the day was divided into three periods: school time (8:00–16:00), after-school time (16:00–24:00), and sleep time (0:00–8:00). These periods were selected based on typical daily routines of local primary and secondary school children. For each participant, the total orthosis-wearing time in each period was calculated for each day. Data from all 30 days for each participant were averaged to obtain the daily wearing time for each period. Peak usage times and durations within these three periods were analyzed to provide an understanding of compliance behavior.

Descriptive statistics, including mean and standard deviation, were calculated for the demographic variables, the average daily wearing time over a month, and for each of the four weeks. A one-way analysis of variance (ANOVA) was used to compare the average wearing times across the three time periods. Post hoc pairwise comparisons were performed using Tukey’s Honestly Significant Difference (HSD) test to identify significant differences between specific periods. Statistical significance was defined as *p* < 0.05. All statistical analyses were conducted using Statistical Package for the Social Sciences (SPSS) software (version 27.0, IBM Corp., Armonk, NY, USA).

## 3. Results

Out of the 14 recruited patients, one had a defective sensor, and another did not wear the orthosis during the first month, leaving data from 12 patients (eight females and four males) eligible for analysis. The average age of the patients was 13.0 ± 0.9 years, the average body mass index was 17.1 ± 2.8 kg/m^2^, and the diagnosed Cobb angle was 28.5° ± 5.2°.

**Figure 3:**
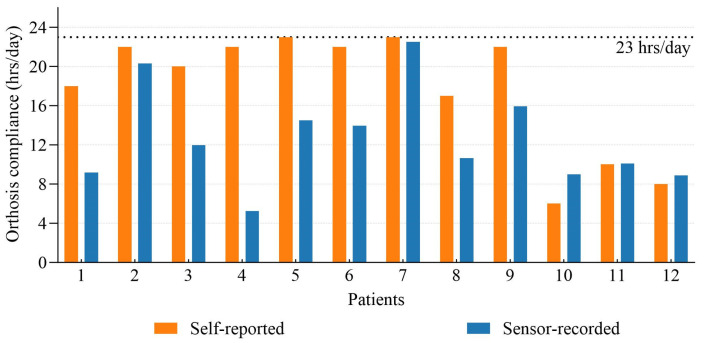
Comparison of self-reported and sensor-recorded orthosis compliance for the twelve patients.

Figure 3 compares self-reported and sensor-recorded average daily wear time in a month. The patient’s estimated average time of wearing the orthosis was 17.8 ± 6.2 h per day (ranging from 6 to 23 h/day), while the sensor data reported an average wearing time of 13.3 ± 5.0 h/day (ranged 5.7 to 22.3 h/day). Most patients overestimated their wearing compliance during the adaptation period.

**Figure 4:**
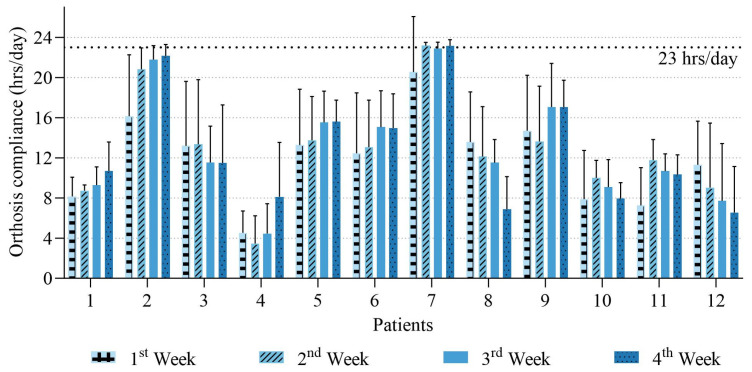
Average weekly wear time for the twelve patients with error bars showing standard deviations.

Figure 4 shows the average daily wear time for each week based on sensor data for twelve patients. Many patients wore the orthosis for less than the recommended 8 h per day in the 1st week, and the increase in wear time over the subsequent weeks did not follow the suggested rate of progression. By the 4th week, most patients did not meet the prescribed target of 23 h per day. Only two patients (patients 2 and 7) almost achieved the target, while others showed significantly lower wear times, with some falling below 20 h.

There were differences in wear time across patients over the four weeks. Some patients (e.g., patient 7) exhibited relatively consistent wear time with minimal fluctuations, while others (e.g., patients 9 and 12) showed greater variability, reflecting irregular wear patterns.

**Figure 5:**
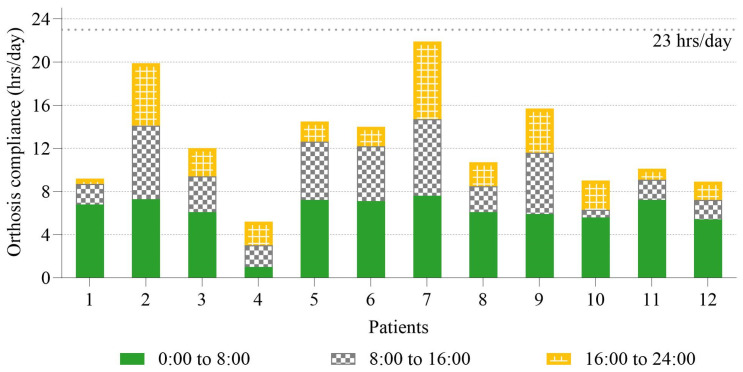
Orthosis compliance for twelve patients during three distinct periods of the day (00:00 to 08:00, 08:00 to 16:00, and 16:00 to 24:00). The total height of each bar represents the total daily wear time, with each color indicating compliance during a specific period.

The daily wearing patterns of the orthosis were also analyzed (Figure 5). The period when patients slept (0:00 to 8:00) was the most common time for wearing orthoses, averaging 6.1 h per day in the first month. The average wearing time for school time (8:00–16:00) and after-school time (16:00–24:00) was 3.7 and 2.8 h per day, respectively. One-way ANOVA revealed a significant difference in orthosis-wearing time across the three periods (F(2,33) = 8.58, *p* < 0.001). Post hoc pairwise comparisons using Tukey’s HSD test indicated that wearing time during sleep was significantly longer than both school time (*p* = 0.016) and after-school time (*p* = 0.001). However, no significant difference was found between school time and after-school time (*p* = 0.549).

## 4. Discussion

This pilot study aimed to evaluate the effectiveness of an integrated force and temperature sensor system for objectively measuring orthosis compliance in the patients with AIS. The results demonstrated that the combined use of force and temperature data provided a more comprehensive understanding of orthosis-wearing behavior compared to patient self-reports. During the one-month adaptation period, most patients overestimated their wear time and primarily wore the orthosis during sleep.

### 4.1. Self-Report and Sensor-Record

The results show that self-reported compliance (17.8 ± 6.2 h/day) was overestimated compared to sensor-recorded wear time (13.3 ± 5.0 h/ day), consistent with previous findings [21,25]. This discrepancy was likely from recall or social desirability bias, where patients may feel comfort in reporting adherence to prescribed wear times even when not fully compliant. Although a study reported only minimal differences (e.g., 0.1 h) between subjective and objective compliance when patients reported daily [22], this method is less practical and efficient for long-term treatment.

A randomized clinical trial demonstrated that patients informed about compliance monitoring showed significantly higher orthosis-wearing compliance compared to those unaware of the monitoring, with compliance rates of 85.7% versus 56.5% during the initial treatment phase [26]. This finding suggests that awareness of being monitored may positively influence patient behavior. Other similar studies also supported this idea [27,28,29]. In our study, however, while patients were aware of the sensor’s presence, they were not informed that it was used to record compliance. This design choice likely minimized behavioral changes caused by monitoring awareness, allowing us to capture more natural adherence patterns. Future studies could investigate strategies such as integrating the monitoring device with a feedback system to further enhance compliance.

### 4.2. Establishing Compliance During the Adaptation Phase

Although patients were instructed to gradually increase wearing time to reach the prescribed 23 h/day by the end of the first month, only two patients achieved this target (Figure 4). These two patients also demonstrated high compliance in the first week, wearing the orthosis for more than 16 h/day. This observation aligns with previous studies, which emphasize that initial compliance during the early stages of treatment is important for establishing adherence and improving the likelihood of treatment success [15,30]. However, many patients failed to meet the recommended 8 h of wear time in the first week, and the subsequent increase in wear time was inconsistent, indicating challenges in achieving the prescribed goals even during the adaptation period.

The stability of daily wear time also varied among patients. Some (e.g., patient 7) maintained consistent daily wear durations, while others (e.g., patients 9 and 12) exhibited large fluctuations (with large standard deviation), reflecting irregular compliance behaviors. Early identification of such non-compliance is critical, as it could allow timely interventions, such as providing additional support or counseling for patients and their families during the adaptation period [29,31,32]. Previous studies have shown that longer and more consistent wear times are associated with better outcomes and reduced scoliosis progression, highlighting the importance of wear compliance for orthotic treatment [8,28,33,34,35]. Additionally, one study found that patients prescribed 19–22 h/day tended to have better adherence compared to those prescribed <19 h/day or >22 h/day [36]. This suggests that slightly adjusting the prescribed target during the early stages of treatment may help improve overall compliance. Future studies are needed to explore the feasibility of this approach in greater detail.

### 4.3. Wear Time Distribution

Previous studies have reported higher compliance during daytime [37,38], while others have focused on nighttime brace use [12,16,39]. In this study, nighttime (0:00–8:00) was the most common time for wearing orthoses, with an average wearing time of 6.1 h per day in the first month. In contrast, the average wearing times during school hours (8:00–16:00) and after-school hours (16:00–24:00) were significantly shorter, at 3.7 and 2.8 h per day, respectively (Figure 5). These findings are consistent with prior reports that nighttime wearing is preferred, as it does not interfere with daily activities, school, or social interactions. In contrast, low wearing time during school hours may be due to social isolation, or practical challenges, such as limited opportunities to adjust the orthosis [40,41]. Similarly, after-school periods may involve physical activities or social interactions that discourage orthosis use.

While nighttime wearing may offer practical advantages, it is important to consider its potential limitations. One study indicated that the pressure exerted by the orthosis on the body is closest to the prescribed load during daytime use, whereas nighttime use exhibits the least effective pressure levels [42]. This suggests that, despite the longer wearing duration at night, the quality of wearing may not meet the required standards. Therefore, improving daytime adherence remains crucial. Targeted interventions such as managing discomfort and visual aesthetics, working with schools to create more supportive environments, or allowing private spaces for donning or adjusting the orthosis, could improve compliance and potentially enhance treatment outcomes [41,43,44].

### 4.4. Strength of Integration of Force and Temperature

The integration of force and temperature sensors represents a significant advancement in compliance monitoring. Previous studies that relied solely on temperature sensors faced challenges, particularly in warmer climates where ambient temperatures can exceed the commonly used thresholds of 28.0–33.0 °C [12,13,16,39]. This limitation is particularly relevant in Hong Kong’s climate, where summer temperatures frequently exceed these thresholds, potentially leading to false-positive readings. The addition of force sensors provides important information on actual orthosis wear, distinguishing between environmental heat exposure and real patient use.

The combination of force and temperature sensors also addresses limitations in scenarios where orthosis tightness decreases over time. Previous studies indicate that the tightness of orthosis fit and consequently the force exerted by the orthosis gradually decreases over time as the patient adapts to wearing it. One study found a 30% reduction in tightness over two weeks, while compliance increased from 7% to 90% [45]. This suggests that the initial target force may not reflect the actual controlling force required or achieved after a period of wear. Fixed force magnitude thresholds alone can underestimate compliance due to fluctuations caused by self-correction exercises or loose orthosis wear [16,18]. During pilot testing, force fluctuation frequency (0.80–0.85 times per minute) was shown to capture natural pressure variations (e.g., breath and self-correction exercise) during wear, providing another method of compliance assessment.

However, this dual-sensor system is not without limitations. In extreme cases, such as prolonged high ambient temperatures or improperly worn orthoses with minimal contact, the combined data from force and temperature sensors may not accurately reflect wear compliance. For example, when the orthosis is loosely worn, the force sensor may detect insufficient contact force, while high ambient temperatures may interfere with the temperature sensor’s ability to distinguish between “worn” and “not worn” states. Despite these challenges, the integrated approach ensures greater accuracy under most conditions and represents a step forward in compliance monitoring. Moreover, the compliance recorded in this study likely reflects more instances of orthosis wear that adhere to the prescribed standards.

### 4.5. Limitations and Future Research

This study has several limitations. First, the small sample size limits the ability to generalize findings. Further research with larger samples and covering the entire treatment period is necessary to fully understand the relationship between compliance and treatment outcomes. Secondly, the weighting between temperature and force data in the compliance calculation requires refinement, as current methods may not fully capture the quality of orthosis use. Additionally, exploring metrics like force amplitude could provide additional insights. Third, the 30-minute stabilization period for sensor data may misclassify short-term wearing patterns, especially during frequent orthosis removal for short breaks. This could introduce errors in compliance estimates. Future studies should optimize algorithms and explore integrating additional sensors, such as inertial measurement units (IMUs) [45], to capture daily activities and postures, providing a more comprehensive understanding of wear behaviors.

## 5. Conclusions

This study introduces a novel method for monitoring compliance in AIS treatment using an integrated force and temperature sensor. The findings suggest that the objective monitoring system can provide more detailed information about orthosis wear compliance, which may enhance the understanding and management of orthotic treatment in patients with AIS. Future studies with large samples and longer durations should aim to refine sensor technology, explore wearing quality, and investigate the correlation between compliance and treatment outcomes over a whole treatment period.

## Data Availability

Data are contained within the article.
